# Future targets for migraine treatment beyond CGRP

**DOI:** 10.1186/s10194-023-01567-4

**Published:** 2023-06-28

**Authors:** Linda Al-Hassany, Deirdre M. Boucherie, Hannah Creeney, Ruben W. A. van Drie, Fatemeh Farham, Silvia Favaretto, Cédric Gollion, Lou Grangeon, Hannah Lyons, Karol Marschollek, Dilara Onan, Umberto Pensato, Emily Stanyer, Marta Waliszewska-Prosół, Wietse Wiels, Hui Zhou Chen, Faisal Mohammad Amin, on behalf of the European Headache Federation School of Advanced Studies (EHF-SAS)

**Affiliations:** 1 Department of Internal Medicine, Division of Vascular Medicine and Pharmacology, Erasmus MC University Medical Center, Rotterdam, The Netherlands; 2 Wolfson Centre for Age-Related Diseases, King’s College London, London, UK; 3 Department of Cardiology, Division of Experimental Cardiology, Erasmus MC University Medical Center, Rotterdam, The Netherlands; 4 Department of Headache, Iranian Centre of Neurological Researchers, Neuroscience Institute, Tehran University of Medical Sciences, Tehran, Iran; 5 Headache Center, Neurology Clinic, University Hospital of Padua, Padua, Italy; 6 Department of Neurology, University Hospital of Toulouse, Toulouse, France; 7 Neurology Department, Rouen University Hospital, Rouen, France; 8 Institute of Metabolism and Systems Research, College of Medical and Dental Sciences, University of Birmingham, Birmingham, UK; 9 Department of Neurology, Wroclaw Medical University, Wrocław, Poland; 10 Spine Health Unit, Faculty of Physical Therapy and Rehabilitation, Hacettepe University, Ankara, Turkey; 11 Department of Clinical and Molecular Medicine, Sapienza University, Rome, Italy; 12 Neurology and Stroke Unit, IRCCS Humanitas Research Hospital, Rozzano, Milan, Italy; 13 Humanitas University, Pieve Emanuele, Milan, Italy; 14 Faculty of Medicine and Pharmacy, Vrije Universiteit Brussel, Brussels, Belgium; 15 Danish Headache Center, Department of Neurology, Faculty of Health and Medical Sciences, Rigshospitalet Glostrup, University of Copenhagen, Copenhagen, Denmark; 16 Department of Neurorehabilitation/Traumatic Brain Injury, Rigshospitalet, University of Copenhagen, Copenhagen, Denmark

**Keywords:** Migraine, Non-CGRP targets, Pituitary adenylate cyclase-activating polypeptide, Vasoactive intestinal polypeptide, Amylin, Adrenomedullin, Nitric oxide, Phosphodiesterases, Ion channels

## Abstract

**Background:**

Migraine is a disabling and chronic neurovascular headache disorder. Trigeminal vascular activation and release of calcitonin gene-related peptide (CGRP) play a pivotal role in the pathogenesis of migraine. This knowledge has led to the development of CGRP(-receptor) therapies. Yet, a substantial proportion of patients do not respond to these treatments. Therefore, alternative targets for future therapies are warranted. The current narrative review provides a comprehensive overview of the pathophysiological role of these possible non-CGRP targets in migraine.

**Findings:**

We covered targets of the metabotropic receptors (pituitary adenylate cyclase-activating polypeptide (PACAP), vasoactive intestinal peptide (VIP), amylin, and adrenomedullin), intracellular targets (nitric oxide (NO), phosphodiesterase-3 (PDE3) and -5 (PDE5)), and ion channels (potassium, calcium, transient receptor potential (TRP), and acid-sensing ion channels (ASIC)). The majority of non-CGRP targets were able to induce migraine-like attacks, except for (i) calcium channels, as it is not yet possible to directly target channels to elucidate their precise involvement in migraine; (ii) TRP channels, activation of which can induce non-migraine headache; and (iii) ASICs, as their potential in inducing migraine attacks has not been investigated thus far.

Drugs that target its receptors exist for PACAP, NO, and the potassium, TRP, and ASIC channels. No selective drugs exist for the other targets, however, some existing (migraine) treatments appear to indirectly antagonize responses to amylin, adrenomedullin, and calcium channels. Drugs against PACAP, NO, potassium channels, TRP channels, and only a PAC_1_ antibody have been tested for migraine treatment, albeit with ambiguous results.

**Conclusion:**

While current research on these non-CGRP drug targets has not yet led to the development of efficacious therapies, human provocation studies using these targets have provided valuable insight into underlying mechanisms of migraine headaches and auras. Further studies are needed on these alternative therapies in non-responders of CGRP(-receptor) targeted therapies with the ultimate aim to pave the way towards a headache-free future for all migraine patients.

## Introduction

Migraine is a chronic neurovascular headache disorder, typically characterized by moderate to severe headache attacks which are accompanied by nausea, vomiting, photo- and phonophobia [1]. Approximately one third of migraine patients additionally suffer from transient neurologic symptoms called migraine auras [1]. Migraine poses a large socioeconomic burden and is ranked as the most disabling disorder in women under the age of fifty [2] — in whom prevalence is the highest [3, 4]. While exact pathophysiological mechanisms remain elusive, increased (sub)cortical excitability, trigeminovascular activation, and release of the neuropeptide calcitonin-gene related peptide (CGRP) — a member of the larger calcitonin family [5] — have been consistently demonstrated to play a pivotal and causative role in the pathogenesis of migraine [6, 7, 8]. The involvement of neuronal mechanisms (e.g. cortical spreading depression and sensitization of perivascular sensory nerve terminals) and structures (e.g. the hypothalamus and brainstem) are crucial for the initiation of migraine attacks, including the premonitory and aura phase [9]. CGRP alone seems unable to either activate or sensitize mechanosensitive meningeal nociceptive neurons in rats [10]. Nevertheless, its discovery has made CGRP the primary pharmacological target of recently approved (preventive) treatments that either target CGRP or its receptor, namely monoclonal antibodies and gepants [11, 12]. Despite their efficacy and tolerability in a substantial portion of both episodic and chronic migraine patients [13, 14], a significant percentage of migraine patients are classified as ‘non-responders’ and exhibit insufficient or no response to these CGRP(-receptor) targeted therapies. This emphasizes the need to explore the potential role of alternative substrates, which might serve as targets for future therapies in a variety of migraine patients [15]. Furthermore, the complex pathogenesis of migraine and its heterogeneous manifestations in patients suggest that different signaling pathways (neuropeptides and neurotransmitters) might be involved in different migraine patients [16] — highlighting the need to further explore these targets. These targets can broadly be categorized into (i) metabotropic receptors or G protein-coupled receptors, which include other members of the calcitonin family of peptides, i.e. amylin and adrenomedullin; (ii) intracellular targets; and (iii) ion channels. In this narrative review, a comprehensive overview is provided of the pathophysiological role of these non-CGRP mechanisms in migraine. We aimed to focus on three main questions for each target, namely whether (i) it has the potential to induce migraine-like attacks; (ii) we have drugs that target its receptors; and (iii) drugs against the substance have been tested for migraine treatment. Our primary aim was to provide a summary of current clinical evidence of these targets, considering that they might serve as a target for future pharmacological treatments and a valuable extension of the current therapeutic armamentarium for migraine.

## Metabotropic receptors (G protein-coupled receptors)

### Pituitary adenylate cyclase-activating polypeptide

**Fig. 1:**
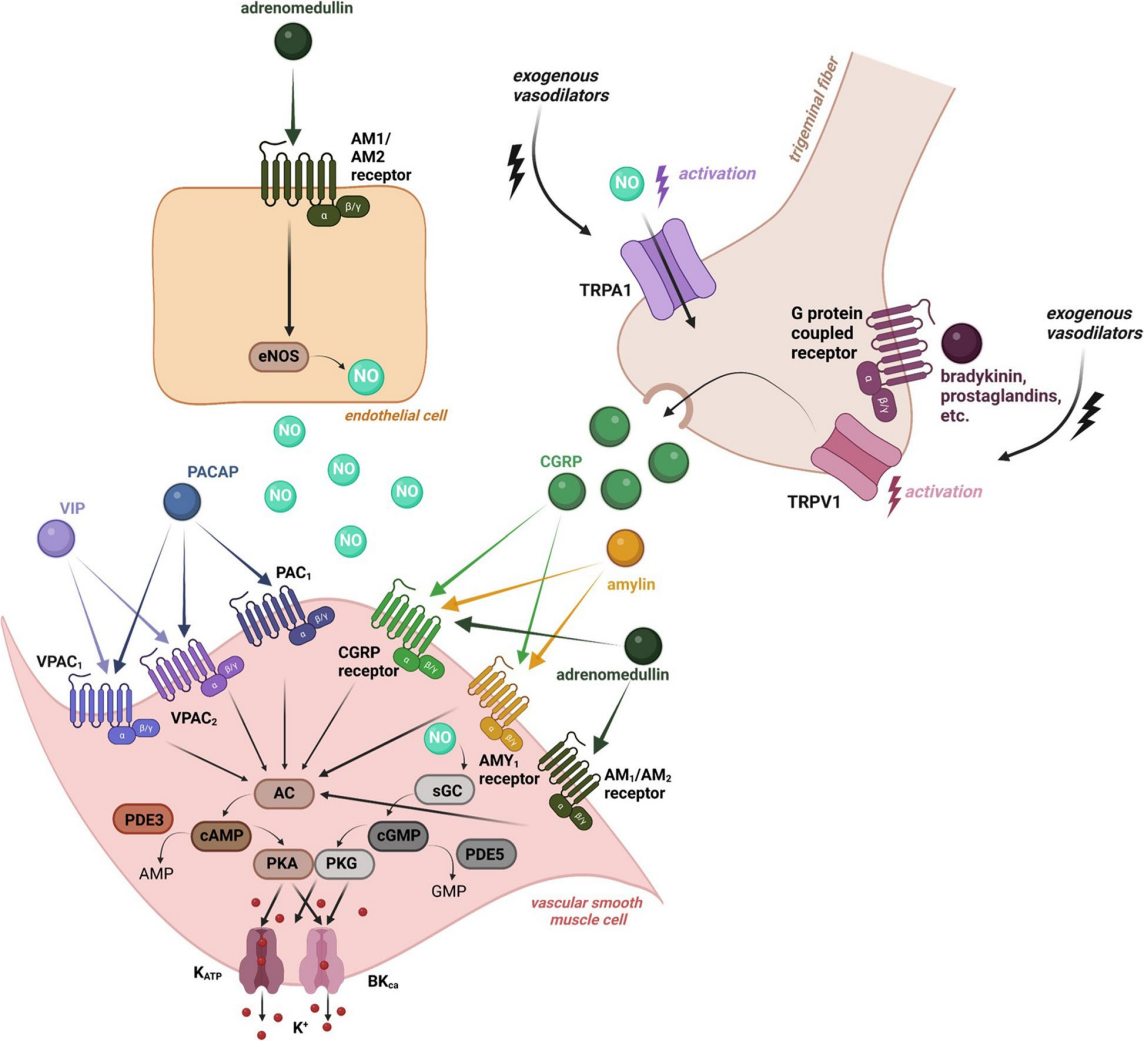
A schematic, yet simplified, overview of (the interaction of) several CGRP-related and non-CGRP-related targets that might be of interest as future therapies in migraine. The final action of these targets is the efflux of potassium via the opening of K_ATP_ and BK_Ca_ channels. The figure was created using *Biorender*. Abbreviations: AC, Adenylate Cyclase; ADM, Adrenomedullin; AMP, Adenosine Monophosphate; AMY_1_, Amylin Receptor 1; AMY_2_, Amylin Receptor 2; BK_Ca_, Big Conductance Calcium-Activated Potassium Channel; cAMP, Cyclic Adenosine Monophosphate; cGMP, Cyclic Guanosine Monophosphate; CGRP, Calcitonin Gene-Related Peptide; sGC, soluble Guanylyl Cyclase; K_ATP_, Adenosine Triphosphate-Sensitive Potassium Channel; NO, Nitric Oxide; PAC_1_, Pituitary Adenylate Cyclase-Activating Polypeptide Type 1 Receptor; PACAP, Pituitary Adenylate Cyclase-Activating Polypeptide; PKA, Protein Kinase A; PKG, Protein Kinase G; PDE, phosphodiesterase; VIP, Vasoactive Intestinal Polypeptide; VPAC, Vasoactive Intestinal Polypeptide Receptor

In 1989, pituitary adenylate cyclase-activating peptide (PACAP) was isolated from the hypothalamus [17] and over the years it has become a key molecule of interest in migraine research [18]. PACAP is an endogenous peptide belonging to the vasoactive intestinal polypeptide (VIP), secretin, and glucagon superfamily of peptides, existing in two major isoforms; a 38-amino acid neuropeptide known as PACAP38 and a shorter 27-amino acid truncated version, known as PACAP27 [17]. PACAP38 is more widely expressed, accounting for over 90% of PACAP in mammalian tissues and is detected in the trigeminal sensory and parasympathetic perivascular nerve fibers [18]. PACAP38 binds with equal affinity to the G-protein coupled receptors VPAC_1_ and VPAC_2_ [19, 20], but it is also capable of binding to a third G-protein coupled receptor, PAC_1_ [21] (Table 1). Activation of these receptors triggers the intracellular cyclic adenosine monophosphate (cAMP) pathway (Fig. 1).

**Table 1:** An overview of the most important characteristics of the individual signal molecules (i.e. the site of origin and synthesis, the main receptors they bind to in humans, their half-lives and whether they can cross the BBB), except for the ions potassium and calcium. We have focused on evidence in human tissue/studies as much as possible and we refer the reader to the Guide to Pharmacology (www.guidetopharmacology.org) for a more detailed overview of the receptor pharmacology. Characteristics of CGRP are added for comparison reasons

**Signal molecule**	**Site of origin & synthesis**	**Main receptor(s)/target(s)**	**Half-life of signal molecule**	**BBB permeable?**
**CGRP**	Multiple sources of CGRP are present (including lymphocytes, monocytes and endothelial cells), but the neuronal sources (perivascular nerve endings) are considered to be the most important source [22]	Human CGRP receptor – with a between 10 and 100-fold higher affinity than for the AMY_1_ receptor (as well as for the AMY_2_ and AMY_3_ receptors) [23]	6.9 ± 0.9 min for the fast decay; 26.4 ± 4.7 min for the slow decay [24]	Possibly, supported by preclinical data showing its contribution to central sensitization [23]
**PACAP**	Central nervous system neurons, but also the peripheral nervous system and several nonneural tissues (such as the adrenal gland, gonads, and immune system), reviewed by [25]	PAC_1_ receptor – with a > 1000-times higher affinity than for the VPAC_1_ receptor or VPAC_2_ receptor [26]	PACAP38: < 5–10 min, reviewed by [27] PACAP27: > 45 min [28]	Yes [29]
**VIP**	Produced by neurons, endocrine and immune cells [30]	VPAC_1_, VPAC_2_ and PAC_1_ receptors, with an approximately 1000-times higher affinity for VPAC_1_ and VPAC_2_ compared to PAC_1_, reviewed by [31]	Less than 1 min [32]	Yes, probably unidirectionally from blood to brain [33]
**Amylin**	In β-cells of the islets of Langerhans in the pancreas, reviewed by [34]	CTR, AMY_1_ receptor (CTR + RAMP1), AMY_2_ receptor (CTR + RAMP2), AMY_3_ receptor (CTR + RAMP3) [34] with roughly overlapping potencies according to the Guide to Pharmacology	Compartment 1: 2.1 ± 0.2 min; Compartment 2: 12.2 ± 1.0 min; Compartment 3: 46.9 ± 6.0 min; Mean residence time: 27.7 ± 2.1 min [35] Compartments 1 and 2 display rapid distribution between vascular and extravascular spaces. Compartment 3 displays a slowly exchanging pool	Yes [36]
**Adrenomedullin**	Mainly expressed by the vascular endothelium and by vascular smooth muscle cells; also been described in neurons of the dorsal root ganglia and trigeminal ganglia [37, 38, 39]	AM1 receptor (CLR + RAMP2), AM2 receptor (CLR + RAMP3), CGRP receptor (CLR + RAMP1) (tenfold less potent than αCGRP) [34], for an extensive overview please refer to [40]	22 ± 1.6 min [41]	Yes, it has also shown to play a role in the regulation of BBB characteristics [42]
**NO**	Synthesized by different isoforms of NOS from L-arginine in various tissues [43]	Soluble guanylyl cyclase [44]	0.09 to > 2 s (extravascular half-life), depending on the oxygen concentration [45]	Yes, as well as NO derived from glyceryl trinitrate, nitroglycerine [46]
**PDE-3**	The expression of PDE-3 depends on its subtype (PDE-3A and PDE-3B), but is generally located in vascular smooth muscle cells, the kidney, and trigeminal ganglia (of rats). PDE-3A is mainly expressed in cardiovascular tissue and platelets, while PDE-3B is expressed in adipocytes, immune cells, and hepatocytes [47, 48]	PDE-3 hydrolyses both cAMP and cGMP [48]	*Not applicable*	Unclear, already distributed within the central nervous system [49]
**PDE-5**	PDE-5 is located in vascular and trabecular smooth muscle cells of various organs (aorta, heart, renal vessels, pulmonary vessels, genital apparatus vessels and brain vasculature). It is localized also in myometral cells, blood cells (megakariocytes and platelets), and trigeminal ganglia (of rats) [47, 50]	PDE-5 specifically hydrolyses intracellular cGMP [48]	*Not applicable*	Unclear, already distributed within the central nervous system [49]
**TRPV agonist capsaicin**	Active component of chilli peppers	TRPV1 channel	24 h upon 3% capsaicin topical administration [51]; 24.9 ± 5.0 min after oral administration [52]; 7.1 ± 3.3 min after intraperitoneal administration in rats [53]. Its bioavailability and implications for drug delivery are reviewed by [54]	Likely [55, 56]

*Abbreviations: AMY*_*1*_ Amylin Receptor 1, *AMY*_*2*_ Amylin Receptor 2, *AMY*_*3*_ Amylin Receptor 3, *BBB* Blood Brain Barrier, *cAMP* Cyclic Adenosine Monophosphate, *cGMP* Cyclic Guanosine Monophosphate, *CGRP* Calcitonin Gene-Related Peptide, *CLR* Calcitonin-Like Receptor, *CTR* Calcitonin Receptor, *NO* Nitric Oxide, *NOS* Nitric Oxide Synthases, *PAC*_*1*_ Pituitary Adenylate Cyclase-Activating Polypeptide Type 1 Receptor, *PACAP* Pituitary Adenylate Cyclase-Activating Polypeptide, *PDE* Phosphodiesterase, *RAMP1* Receptor Activity-Modifying Protein, *TRP* Transient Receptor Potential, *VIP* Vasoactive Intestinal Polypeptide, *VPAC* Vasoactive Intestinal Polypeptide Receptor

Intravenous infusion of PACAP38 [57, 58] as well as PACAP27 [59] induced mild and short-lasting headaches in healthy volunteers. In patients with migraine without aura, intravenous infusion of both peptides additionally induced delayed migraine-like attacks. In double-blind, placebo-controlled, crossover trials, 58% of the patients reported migraine attacks after PACAP38 [57] and 55% after PACAP27 [60].

Given the interest in PACAP as a potential target for migraine therapeutics, several studies were conducted to investigate the migraine-evoking properties of PACAP. Preclinical studies indicated that PACAP-specific active transport systems exist across the blood brain barrier (BBB). However, once transported across the BBB, both isoforms are either rapidly degraded or diffuse back across the BBB into the blood, suggesting that PACAP exerts its effects mainly via peripheral mechanisms [61]. *In vitro* studies reported that PACAP38 was able to relax the vascular smooth muscle cells after abluminal, but not after luminal application in cerebral arteries [62]. *In vivo* studies on cerebral hemodynamics demonstrated no effect of intravenous infusion of PACAP38 on regional cerebral blood flow [63]. Moreover, *in vivo* studies using high-resolution magnetic resonance angiography reported a selective, marked and long-lasting vasodilatory effect of intravenous infusion of PACAP38 [20] and PACAP27 [59] on the extracerebral, but not the middle cerebral arteries. Upon administration of sumatriptan, the headache symptoms were relieved alongside contraction of the middle meningeal artery, but not the middle cerebral artery [58]. These findings suggest that the migraine-inducing effect of at least PACAP38 may be outside of the BBB. The mechanism of action is more complex and involves mast cell degranulation [31] and/or prolonged vasodilation [20]. Degranulation of mast cells causes release of histamine. It has been shown in rats that PACAP38 induces histamine release [64], and in migraine without aura patients, histamine triggers migraine-like attacks [65]. Yet, pretreatment with clemastine, a histamine (H1) antagonist, did not reduce the frequency of PACAP38-induced migraine attacks, suggesting an alternative mechanism for the migraine symptoms [66]. Using a double-blind, crossover design, a head-to-head comparison study of PACAP38 and VIP reported a significantly higher migraine-induction rate after PACAP38 compared to VIP (73% vs 18%, respectively). The study also showed prolonged meningeal artery dilation after PACAP38 but not after VIP infusion [20]. Moreover, sumatriptan was able to prevent PACAP38-induced migraine attacks, potentially via modulation of nociceptive transmission within the trigeminovascular system [67]. Thus, the prolonged meningeal artery dilation may play a role in PACAP38-induced migraine attacks. Receptor-wise, the main difference between PACAP38 and VIP is the over 1000-fold higher affinity of PACAP38 to the PAC_1_ receptor (Table 1), which positions the PAC_1_ receptor as a potential novel target for migraine treatment [68]. This led to the development of AMG301, a human monoclonal antibody selective for inhibition of the PAC_1_ receptor. However, clinical trials on AMG301 failed to prevent migraine [69]. Following the lack of success in targeting the PAC_1_ receptor, antibodies targeting the PACAP ligands have been proposed. For further details on the role of PACAP (blockade) in headaches, including migraine, we refer the readers to (recently published) reviews [70–72].

### Vasoactive intestinal polypeptide

VIP is a 28-amino acid peptide with a short half-life of about one minute that is expressed in the intestine, pancreas, tongue, adrenal glands, urogenital tract, and brain (Table 1). VIP is thought to be involved in thermoregulation, cell proliferation, immune response, smooth muscle tone, and nociception [32, 73]. Among the complex scenarios of the trigeminovascular activation in the migraine attack, VIP seems to play an important role along with its homologous peptide PACAP [74]. VIP is released by parasympathetic fibers and exerts its role through the aforementioned receptors VPAC_1_ and VPAC_2_, which show a similar affinity to VIP and PACAP. VIP is theoretically able to bind also to PAC_1_; however, this latter receptor has a 1000-fold higher affinity to PACAP and is, therefore, considered to be PACAP-specific [31, 73] (Table 1, Fig. 1). In recent years, the role of VIP in migraine attacks has been extensively studied. Levels of plasma VIP have been found to be increased in the interictal phase in both adults with episodic and chronic migraine versus controls [75]. Levels of VIP in blood and saliva, though elevated during a spontaneous migraine attack, undergo a significant reduction after triptan administration in adults with migraine [76]. VIP is also involved in craniocervical vasomotor responses, and has a strong vasodilatory effect [76].

In a double-blind placebo-controlled crossover study, Hansen et al. evaluated twelve healthy subjects who received intravenous VIP or placebo over 25 min, which induced a very mild and short-lasting headache in five patients (42%) during VIP infusion compared to only one patient during placebo infusion [77]. In another study performed by Pellesi et al. twelve healthy volunteers were subjected to continuous VIP infusion over two hours, which provoked mild headache in 67% of participants, mainly in the post-infusion period. Interestingly, 25% of the participants reported migraine-like attacks [78]. Rahmann et al. were the first to evaluate the headache occurrence and vasomotor response in migraine patients after infusion of VIP or placebo in a double-blind crossover study [79]. Patients who received VIP showed marked but short-lasting vasodilation of both intracranial and extracranial arteries and a mild immediate headache with respect to placebo but no migraine-like attacks [79]. A more recent study reported that a two-hour infusion of VIP induced migraine attacks in 71% of patients with a history of migraine without aura [80]. The only methodological difference between Rahmann et al. [79] and the recent study from Pellesi et al. [80], was the duration of VIP infusion (i.e., 25 min versus 2 h). This suggests that the long-lasting (probably vascular) effect of VIP is more important for migraine induction. Indeed, the throbbing headache in migraine most likely originates in sensory fibers that transmit pain signals from intra- and extracranial (vasodilated) vessels, in particular arteries [81, 82]. So far, selective blockade of VIP has not been studied as a treatment of migraine. As current results suggest that a prolonged vasodilation due to VIP might provoke migraine-like attacks, VIP blockade might be a potential target in migraine treatment.

### Amylin

Amylin belongs to the calcitonin peptides superfamily that includes CGRP, calcitonin, adrenomedullin, and adrenomedullin 2/intermedin [21, 37]. This is a 37-amino acid peptide structurally related to CGRP and is mainly released by the beta cells of islets of Langerhans in the pancreas [83] (Table 1). Amylin, which is secreted along with insulin, is involved in meal-ending satiation and inhibits insulin secretion [83, 84]. A key function of amylin is to maintain glucose homeostasis and reduce the uptake of glucose through its actions on the secretion of glucagon, gastric emptying, and caloric intake (reviewed by [85]). CGRP and amylin share CGRP and amylin 1 (AMY_1_) receptors, which have a similar structure composed of receptor activity modifying protein 1 (RAMP1) in combination with either calcitonin receptor (CTR) for AMY_1_, or calcitonin receptor-like receptor (CLR) for the CGRP receptor [5] (Table 1). As a result, the investigations of distribution and action of AMY_1_ have been challenged by its cross-reactivity with the CGRP receptor [5] (Fig. 1). While CGRP has equal affinity for the CGRP receptor and AMY_1_, amylin has a much lower affinity than CGRP for the CGRP receptor [83]. Considering these limitations, AMY_1_ has been posited to be distributed on structures involved in migraine pathophysiology, such as the trigeminal ganglion (TG) and the spinal trigeminal complex [83].

The development of the amylin analog pramlintide has aided research into the involvement of amylin in migraine pathophysiology. Pramlintide is an antidiabetic drug, approved by the U.S. Food and Drug Administration, with headache as a side effect in 13–17% of its users [83, 86]. In clinical trials, headache incidence was higher after pramlintide compared with placebo [83]. In coherence, intravenous infusion of pramlintide induced headache in 17% vs 6% with placebo in healthy subjects [87]. A subsequent provocation trial aimed to investigate the migraine induction rate of the pramlintide. In this head-to-head comparison study between pramlintide and CGRP in 36 patients 88% developed headache. Additionally, a migraine-like attack was triggered in 14 patients (41%) after pramlintide and 19 patients (56%) after CGRP. There was no statistical difference between the induction rates as well as the attack phenotypes were similar on both experimental days [88]. Human pharmacology studies demonstrated that one or more of the CTR/RAMP complexes (AMY_1_, AMY_2_, and AMY_3_ receptors) likely mediate the pramlintide-induced effects and migraine-like attacks, rather than CLR-based receptors (namely, the CGRP receptor or adrenomedullin-responsive receptors (AM_1_, AM_2_), which are described in the next section) [88]. Noteworthy, pramlintide produced little arterial vasodilation compared to CGRP (superficial temporal artery dilation, mean maximum change from baseline approximately 8.5% versus 115%, respectively), which could be explained by its lower potency compared to CGRP (> 1000-fold lower potency) for the CGRP receptor and possibly by a different distribution of CTR- and CLR-coupled receptors in the vascular tissue [88]. Further studies showed an increased amylin blood level in chronic migraine with basal level of pain, but not in interictal episodic migraine, suggesting that amylin may serve as a diagnostic biomarker for chronic migraine [89].

There is currently no treatment available to specifically antagonize the AMY_1_ receptor, although CGRP receptor-targeting treatments erenumab and rimegepant have been shown to antagonize AMY_1_ with a lower affinity compared with the CGRP receptor [90–92].

### Adrenomedullin

Adrenomedullin is a 52-amino acid peptide belonging, like amylin, to the calcitonin peptide superfamily. It shares several structural features with CGRP such as a C-terminus amide and a loop structure in the N-terminus [93]. The adrenomedullin receptors AM_1_ and AM_2_ have a structure composed of RAMP2 or RAMP3, respectively, in combination with CLR. Interestingly, adrenomedullin acts on the CGRP receptor as well but is ten-fold less potent than CGRP [40] (Table 1). While CGRP and adrenomedullin are both released by sensory C-fibers, adrenomedullin is highly expressed by the vascular endothelium in contrast to CGRP [37, 94] – in particular within the cerebral circulation [38] (Fig. 1). Adrenomedullin stimulates endothelial-induced vasodilation within the cerebral circulation and plays an important role in the regulation of the BBB [95]. Nevertheless, the vasodilatory and hypotensive ability of adrenomedullin is less potent compared with CGRP. Expression of adrenomedullin and its receptors has also been described in neurons of the dorsal root ganglia and TG [37, 39], suggesting a role for adrenomedullin in the pain pathway. With a widespread distribution in various tissues, adrenomedullin plays a role in cardioprotection, reproduction, renal function, and lymphatic system [96]. Its anti-inflammatory, anti-apoptotic, and proliferative properties give adrenomedullin a potent protective role. This raised the interest for its therapeutic applications, notably in pulmonary hypertension and acute myocardial infarction (reviewed by [97]). The cross reaction of adrenomedullin with CGRP has spiked interest in its involvement in migraine pathophysiology. In mice, intrathecal administration of adrenomedullin induced mechanical hyperalgesia and inflammatory pain [98].

Two clinical studies explored the ability of adrenomedullin to provoke a migraine-like attack. A recent placebo-controlled two-way crossover study showed that adrenomedullin administration in 20 migraine patients induced a migraine-like attack in 55% of patients compared with 15% after placebo [99]. Conversely, twelve migraine patients showed in another study the same frequency (33.3%) of migraine-like attack after adrenomedullin administration compared with placebo, but the amount of adrenomedullin infused was slightly lower compared with the aforementioned study [100]. Finally, adrenomedullin was associated with headache induction in other human studies designed to explore the vascular effects of adrenomedullin [5]. Hence, adrenomedullin might induce migraine at possibly supraphysiological concentrations, by also activating the CGRP receptor, but the possibility of the occurrence of an attack at physiological concentrations is unclear to the best of our knowledge. A previous study in migraine patients showed lower plasma levels of adrenomedullin in the ictal and interictal phase compared to controls, suggesting an imbalance between CGRP and adrenomedullin in the migraine pathophysiology [101]. Moreover, which adrenomedullin-responsive receptors (AM_1_, AM_2_) or the CGRP receptor mediate these migraine-like responses have yet to be elucidated [99]. AM_22-52_ is the only adrenomedullin antagonist available but seems to be limited by its weak antagonizing effect on adrenomedullin responses (including adrenomedullin-stimulated cAMP) in rat cells, although it has been shown to have CGRP inhibiting effects [102, 103]. There is currently no treatment available to specifically antagonize adrenomedullin or its receptors, although one *in vitro* study demonstrated that the CGRP-receptor targeting monoclonal antibody erenumab and the CGRP-receptor small-molecule antagonist telcagepant antagonized not only CGRP, but also adrenomedullin signaling at the CGRP receptor [104].

## Intracellular targets

### Nitric oxide

Nitric oxide (NO) is a free radical synthesized by isoforms of nitric oxide synthase (NOS): endothelial NOS (eNOS), predominantly expressed in the endothelium and exerting a vasodilating effect; neuronal NOS (nNOS), present in central and peripheral nervous system; and inducible NOS (iNOS), which is involved in the innate immune system [46]. Upon binding of NO to its intracellular receptor, soluble guanylyl cyclase (sGC), levels of the second messenger cyclic guanosine monophosphate (cGMP) increase, and this leads to the opening of adenosine triphosphate-sensitive potassium (K_ATP_) channels and big conductance calcium-activated potassium (BK_Ca_) channels, which are speculated to play a crucial role in generating migraine attacks [105, 106] (Table 1, Fig. 1). The NO donor nitroglycerin, also known as glyceryl trinitrate, is one of the most commonly used experimental triggers for migraine headache [107]. Both headache of a pulsating nature during the administration of the drug and headache with a delayed onset of action were observed, fulfilling the characteristics of migraine without aura. There is also an increased sensitivity to nitroglycerin among migraine patients compared with individuals without a history of headache [108].

There have been studies towards new drugs targeting the arrest of the NO-cGMP cascade. An experiment in mice demonstrated that administration of the sGC stimulator VL-102 produced acute and sustained hyperalgesia. This effect was blocked by an sGC inhibitor (ODQ, 1H-[1,2,4]oxadiazolo[4,3,-a]quinoxalin-1-one), but also by several antimigraine medications (sumatriptan, topiramate, and propranolol) [109].

Additionally, researchers have explored NOS inhibition. Non-selective inhibitors may have important limitations mainly because of the significance of eNOS in regulating blood pressure [110], and therefore, researchers have focused on selective drugs. In a study of seven patients with migraine without aura, a significant increase of iNOS was observed during unprovoked migraine attacks [111]. Nevertheless, the highly selective iNOS inhibitor GW274150 failed to show efficacy both in acute [112] and preventive [113] treatment. In another study NXN-188, a combined nNOS inhibitor and serotonin (5-HT_1B/1D_) receptor agonist, inhibited the induced release of immunoreactive CGRP (iCGRP) from rat dura mater, TG, and trigeminal nucleus caudalis (TNC). Nevertheless, NXN-413, a selective nNOS inhibitor, inhibited iCGRP release from dura mater, but not from the TG and TNC in the same study [114]. NXN-188 did not reach a significant advantage over placebo in terminating migraine attacks in a small (*n* = 50) randomized trial [115]. Despite the ambiguous data concerning NOS inhibition as potential therapeutic target, the evidence of involvement of NO-cGMP cascade in the pathophysiology of migraine is constantly growing. Hence, there is a need for more reliable clinical trials, possibly on larger samples, to identify novel drug candidates targeting this molecular pathway.

### Phosphodiesterase-3

CGRP activates adenylate cyclase transmembrane receptors in cerebral vascular cells and increases the second messenger cAMP. Intracellular accumulation of cAMP induces migraine attacks [116]. Phosphodiesterase-3 (PDE3) degrades cAMP (Fig. 1). In rats, PDE3 and cyclic nucleotide-gated ion channels are expressed in the trigeminovascular system, which includes the middle cerebral artery, basilar artery, TG, and dura mater [117]. Therefore, modulation of PDE3 in cAMP levels and activation of cyclic nucleotide-gated ion channels may play a role in the pathogenesis of migraine-like attacks [116]. PDE3 is indeed expressed in the TG, vascular smooth muscle cells, and cerebral arteries [118] (Table 1). Trigeminal neurons have been shown to be sensitized through an increase of cAMP [119, 120]. PDE3A and B are found with CGRP in the neuronal part of the trigeminovascular system [116]. Also, PDEs 3 and 4 degrade cAMP in vascular smooth muscle cells [121].

Since cilostazol inhibits PDE3, intracellular cAMP accumulates and the opening of K_ATP_ channels is facilitated, causing vascular smooth muscle relaxation and dilatation of cerebral arteries [122, 123] without affecting regional blood flow [116, 121, 124]. Thus, cilostazol leads to activation of the pathway that is further downstream than CGRP and PACAP (Fig. 1), and has a high success-rate to induce migraine-like attacks in 86% of adult patients with migraine without aura [116, 120, 125, 126], but also in healthy individuals [121]. Such is its potential role in migraine headache: modulating nociceptive input in regions where pain is processed in the neuronal parts of the trigeminovascular system [125]. These observations highlight the possible role of PDE3 in pain pathways involved in migraine headache [120, 125]. The effects of sumatriptan were studied in a cross-over study by Falkenberg et al. including migraine without aura patients in whom cilostazol-induced migraine was provoked. The majority of these patients were triptan responders, and the reduction in headache severity in the group given oral 50 mg sumatriptan was not significant at two hours (primary end-point), but rather at four hours when compared with the placebo group [118]. The authors concluded that, therefore, no clear effect was observed of sumatriptan on reducing cilostazol-induced migraine attacks. Hereafter, a double-blind cross-over study was conducted to study migraine headache development after cilostazol administration in migraine without aura patients [125]. The reduction of headache intensity in the group given 6 mg subcutaneous sumatriptan was significant at the second and fourth hour compared with the placebo group [125]. These results indicate that subcutaneous treatment rather than oral administration with sumatriptan is more effective in these cilostazol-induced headaches. These differences are probably related to its different pharmacokinetic properties, namely the faster peak in plasma concentrations, although a stronger placebo effect cannot be ruled out [125]. Khan et al. administered oral and subcutaneous sumatriptan to patients who developed migraine-like headaches after cilostazol administration. They stated that the highest decrease in headache score was after subcutaneous administration, whereas there was a smaller decrease in oral sumatriptan and other rescue drugs [120].

Thus, the modulation of PDE3 in intracellular cAMP levels may play a role in migraine attacks. Subcutaneous sumatriptan rather than oral sumatriptan may be an effective treatment modality for cilostazol-induced migraine attacks and headache intensity, although both exert relatively modest effects. Further studies are warranted to elucidate whether the modest extracellular effects of sumatriptan are due to its inability to completely block intracellular responses, namely the increased cAMP levels, induced by cilostazol [118].

### Phosphodiesterase-5

Phosphodiesterase-5 (PDE5) is an intracellular enzyme that breaks down cGMP. Inhibition of PDE5 increases intracellular cGMP levels, leading to smooth muscle cell relaxation and neuronal stimulation [127] (Table 1, Fig. 1). Conversely, CGRP acts via increasing intracellular cAMP levels. PDE5 inhibitors, such as sildenafil and tadalafil, were initially produced for cardiovascular disorders, however, they were later demonstrated to be effective for sexual impotence and to have headache-inducing capacities [127, 128]. Indeed, several experimental studies revealed that up to 83% of migraine patients experience migraine-like headaches after sildenafil administration [129], and up to one third of individuals without migraine may also experience headaches [130].

CGRP and sildenafil act on cAMP and cGMP intracellular signaling pathways, respectively [131]. Nonetheless, patients experience migraine attacks with overlapping clinical features after administration of CGRP and sildenafil [131] suggesting that these two intracellular pathways likely converge in a downstream common denominator responsible for the biological migraine initiating cascade. Accordingly, mounting preclinical evidence supports a reciprocal influence between cAMP and cGMP pathways [47, 131–133]. Notably, administration of sildenafil induced more attacks in migraine patients compared to CGRP (89% vs 67%), potentially reflecting a more potent migraine-induction capacity [131].

Whilst the intracellular culprits of PDE5 inhibitors, and headache-inducing substances at large, have been recognized, their biological effects and site of action within the nervous system are still not fully understood. Initial investigations focused on potential cerebrovascular changes mediated by these substances. One study found no large intracranial or extracranial artery dilation after administering PDE5 inhibitors [130]. Another study observed dilation of intradural and extradural middle meningeal artery after CGRP or PDE5 inhibitor infusion, yet with no direct temporal correlation between arterial dilation and pain, suggesting this may reflect the activation of perivascular dural afferents [134]. Therefore, sensitization of perivascular sensory nerve terminals represents arguably a critical biological mechanism of migraine attack initiation [129]. Additionally, a transient increase in glutamate levels in the brainstem has been observed exclusively after PDE5 inhibitor administration, suggesting a possible different effect than CGRP [135]. Yet different pharmacokinetics properties of these molecules, namely the impermeability of the BBB to CGRP, but not to PDE5 inhibitors, may explain these results without discarding the common signaling pathway hypothesis.

PDE5 modulation represents a future unexplored therapeutic landscape. However, at present, no specific drugs which aim to increase PDE5 levels have been investigated in migraine. Thus, emulating the virtuous bench-to-bedside journey of the CGRP system may ensure further outstanding achievements in migraine.

## Ion channels

A large range of ion channels are involved in migraine, likely including specific potassium channels and calcium channels. Furthermore, transient receptor potential (TRP) channels and acid-sensing ion channels (ASICs) have been proposed to play a role in migraine, due to their important roles in the trigeminothalamic and nociception systems. Yet, considering the fact that the conduction of human provocation models to study the specific activation of ASICs in humans is lacking, we consider them as two promising targets for future research (Box 1).

### Potassium channels

Potassium channels are membrane-spanning protein complexes that are involved in the transportation of potassium ions to mediate or increase the membrane potential. Their activation is dependent on the regulatory domain differentiating the large number of potassium channels. Opening of K_ATP_ channels causes relaxation of vascular smooth muscle cells by K^+^ efflux, a reversible hyperpolarization of the membrane, and decreased intracellular Ca^2+^ [123, 136]. These phenomena can consequently lead to the induction of a headache attack [137, 138].

High extracellular concentrations of potassium can trigger cortical spreading depression (CSD), which is thought to be a cause of aura in migraine with aura patients [139, 140], activating an inflammatory cascade, which leads to an increase of intracellular calcium and release of proinflammatory peptides (e.g. CGRP).

Genome-wide association studies in migraine patients identified several potassium channel linked genes that were likely susceptible, providing further evidence for their involvement (reviewed in [141]). Mutations in the two-pore-domain potassium channel TRESK have been identified in migraine patients. TRESK inhibits TREK1 and TREK2, thereby increasing excitability of the TG [142]. Reducing the TG excitability using the TREK1/TREK2 agonist ML67-33 reduced an NO donor-induced migraine-like phenotype in mice in a similar manner as the CGRP receptor antagonist olcegepant. Moreover, it completely reversed TG-mediated NO donor-induced facial allodynia in rats [143].

Acting by direct opening of K_ATP_ channels, levcromakalim is a potent vasodilator as well as a trigger of headache in healthy subjects and of migraine-like attacks in migraine patients following systemic administration [136, 144]. Some observations revealed that levcromakalim acts downstream from the CGRP receptor and hypersensitivity caused by levcromakalim is independent of direct CGRP release from the TNC and the TG [136, 139]. On the other hand, experimental mice models showed that levcromakalim-induced hypersensitivity was blocked by CGRP neutralization [123].

*In vitro* studies showed that glibenclamide, a non-specific K_ATP_ channel blocker and a widely used anti-diabetic drug, attenuated PACAP-induced dilation of rat cerebellar and human pulmonary arteries [144, 145].

In healthy volunteers, treatment with glibenclamide did not mitigate PACAP38-induced headache and vascular changes [136]. A randomized, placebo-controlled cross-over study in healthy individuals showed that administration of glibenclamide did not induce headache, nor did it prevent headache or changes in blood pressure and heart rate induced by levcromakalim. Yet, the authors found that glibenclamide rather delayed the onset of the induced headaches [146]. More selective K_ATP_ channel blockers are needed to clarify potential mechanisms of the K_ATP_ channel in pathways involved in migraine, including the PACAP38 signaling pathway.

In contrast to K_ATP_ channels, the specific voltage-gated potassium Kv7 channels potentially reduce migraine when opened, as demonstrated in male rats. The Kv7 channel opener retigabine significantly reduced basal and TRP channel-induced CGRP release *in vitro* [147].

Another investigative target includes the BK_Ca_ channels, which are widely expressed in the brain and cardiovascular system and facilitate potassium efflux, hyperpolarization, and thus decreased neuronal excitability [148, 149]. BK_Ca_ channels require both membrane depolarisation and binding of Ca^2+^ for opening, and have been found to be co-localised with voltage-gated Ca^2+^ channels [150]. Of particular interest here is their role in the activation of vascular smooth muscle cells and related involvement in the trigeminovascular system [151]**.** Additionally, they modulate neurotransmitter release in peripheral neurons, and their opening has been shown to inhibit Aδ-fiber firing in the rat’s TNC when applied directly [152], whereas genetically ablating them from sensory neurons in mice increased nociceptive behaviors in some but not all pain models [153]. BK_Ca_ channels have furthermore been connected with various signaling pathways and factors known to contribute to migraine, such as CGRP and PACAP [154]. This does indeed raise the possibility of downstream involvement in the migraine pathophysiology.

Results of a small pilot study confirmed that pharmacologic opening of BK_Ca_ channels using a small vasoactive molecule (MaxiPost) did indeed provoke cephalic vasodilation in 90% of healthy subjects, as compared to 30% with placebo injection [155]. Finally, Al-Karagholi et al. demonstrated that MaxiPost induced migraine-like attacks – and not merely vasodilatory headache – in 21 of 22 migraine patients [156].

Both preclinical and clinical findings, albeit contrasting, might reflect the wide diversity of BK_Ca_ channel types [148, 150]. Therefore, molecules selectively targeting the channels or specific protein subunits of BK_Ca_ that are involved in migraine pathophysiology should be preferably developed. This in order to avoid non-selective action and related side effects, as is the case with calcium channel blockers and other drugs currently used for migraine therapy [157]. These findings, analogous to earlier research on CGRP, suggest that BK_Ca_ channel antagonists could prove to be promising molecules in the pharmacotherapy of migraine.

### Calcium channels

Calcium channels are voltage-gated ion channels that regulate the flow of Ca^2+^ ions, an essential intracellular second messenger. Calcium blockers have long been used as migraine prophylactic drugs that target broad calcium receptors such as nimodipine and flunarizine that, following the cerebral hypoxia hypothesis, aim to reduce excessive intracellular influx of calcium ions that results from increased neuronal excitability [158, 159]. It should, however, be noted that the efficacy of these drugs is likely not mediated by calcium channel action but by other mechanisms, including the suppression of CSD [160].

One of the main calcium channels implicated in migraine is the voltage-gated Ca_v_2.1, found in presynaptic terminals throughout the central nervous system with its main role involving neurotransmitter release and dendritic transients [161, 162]. These channels give rise to P/Q-type calcium currents and its encoding gene, CACNA1A, stands as a classical genetic locus associated with the rare genetic disease familial hemiplegic migraine (FHM) [163].

Several missense mutations have been found in FHM families, including those that code for the voltage sensor and pore structures in the α_1_ subunit of the channel [163–165]. There is evidence that these mutations present gain-of-function changes in cortical excitatory neurotransmission, reducing the threshold for CSD, all the while interneuron inhibition remains intact, potentially driving an excitatory-inhibitory imbalance [166, 167]. Additionally, this mechanism may also play a role in descending pain modulatory pathways, as its blockade has been shown to enhance trigeminal transmission in the rat [168]. Furthermore, Chan et al. [169] assessed trigeminovascular activity in a genetic mouse model of FHM and found responses that reflect desensitization of CGRP receptors. However, most (experimental) treatments for CACNA1A-associated disorders do not specifically target this channel. One possible exception is 4-aminopyridine; more widely known as a potassium channel blocker, pharmacologic activity on calcium channels has been described [170]. To our knowledge, no clinical studies on migraine using this molecule have yet been carried out. Nonetheless, in the context of CACNA1A-associated spinocerebellar ataxia type 6, some preliminary and preclinical possibilities for selective small molecules were reported in 2018 [171].

The role of calcium channels in migraine has been explored to a limited extent, due to its many roles in neurotransmitter release and secondary messenger pathways, which makes it difficult to extract its precise involvement in migraine. Further work on calcium channels and how they interact with other neurotransmitter circuits, especially in the case of genetic migraine, may uncover new potential therapeutic targets of migraine pathophysiology.

### Transient receptor potential (TRP) channels

The TRP channel superfamily consists of non-selective cation channels that mediate the transmembrane flow of calcium and are mainly implicated in sensory transduction. They are also thought to modulate pain processing and particularly the vanilloid TRP (TRPV) channel subfamily is considered relevant here [172, 173]. Several TRP channels have been identified as potential antimigraine targets, including the TRPV1 and TRP ankyrin 1 (TRPA1) channels [174] (Fig. 1). Briefly, TRPV1 channels process thermal and pH-related nociception, are involved in calcium release in sensory neurons, and are expressed in the trigeminal pathway [175, 176]. Their relevance in migraine extends further, as activation of these channels induces release of CGRP and increases TG activity, which is reversed by sumatriptan [175, 177]. TRPV1 channel modulators have had moderate success in the treatment of migraine, with part of their antinociceptive activity being affected by topical agonists, such as capsaicin, to desensitize the channel [178]. Capsaicin is a TRPV1 agonist and its intranasal administration, as well as its synthetic isosomer civamide, have shown some efficacy for the treatment of cluster headache and migraine [179, 180]. However, discrepancies in efficacy and safety between preclinical and clinical studies justify the need for further research [175].

On the other hand, the TRPA1 channel has attracted attention due to its ability to transduce a multitude of chemical agents such as formaldehyde and cigarette smoke, many of which are also common migraine triggers [174]. Notably, the “headache tree” extract, umbellulone, is able to trigger headache when inhaled, and has shown to act on the trigeminovascular system to induce CGRP release from trigeminal afferents in the dura [181]. Parthenolide, another herb extract, is a partial TRPA1 agonist and has the ability to desensitize it, effecting anti-migraine responses on CGRP-mediated trigeminal activity [182], and positioning it as a potential migraine target. We refer the readers to several excellent reviews on TRP channels for further details on their role in the pathophysiology of migraine [183, 184].

Box 1: ASICs ASICs are voltage-insensitive cation channels widely expressed throughout the nervous system. Four different members have been described (ASIC1-4). ASIC1-3 are pH sensitive, each opening at a specific pH range. Decreased extracellular pH as well as ASIC activation may play a role in migraine. Most ASICs are expressed on primary sensory neurons, however ASIC3 is highly expressed in peripheral neurons. Given that NO donors can potentiate ASIC3 and increase acid-evoked pain [185], its inhibition was tested using a rat model. The ASIC3 blocker APETx2 inhibited durovascular-evoked and NO-induced sensitisation of trigeminal nociceptive responses [186]. Specific blockade of ASIC1, the most predominantly expressed ASIC in the central nervous system, using black mamba venom derived mambalgin-1 or tarantula venom derived PcTx1 attenuated pain signaling in the lower spinal cord, and might also be relevant in other sites such as the trigeminal system [187]. ASICs might contribute to altered activity in the hypothalamus, CSD and sensory input from meninges, therefore ASICs have also emerged as new potential therapeutic targets for migraine [188] (and reviewed in [187]). In order to block all ASICs and be most effective the pharmacological agents might need to gain access to the central nervous system [187]

## Future directions

**Fig. 2:**
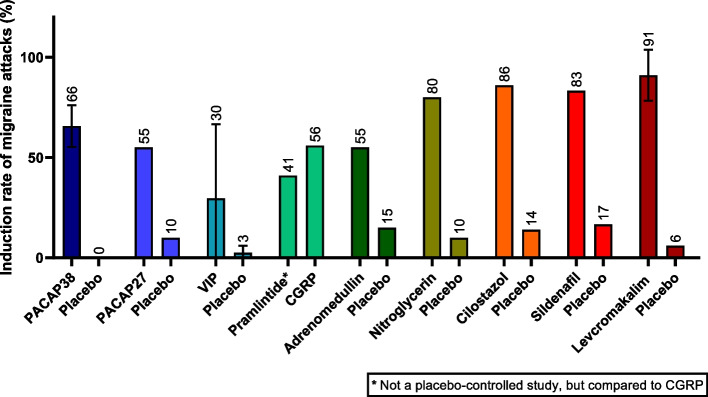
Mean induction rates (with their standard deviations) of migraine attacks (irrespective of aura symptoms) of non-CGRP targets compared to placebo, as observed in placebo-controlled studies in migraine patients. PACAP38 rate is based on [57] and [20] – the latter is a head-to-head comparison study of PACAP38 and VIP (instead of placebo); PACAP27 rate is based on [60]; VIP rate is based on [20] with an active control (PACAP38) and [79, 80]. Please note that infusion duration in the study of Rahmann et al. [79] was 25 minutes, while it was two hours in the study of Pellesi et al. [80]; pramlintide rate is based on [88] which was compared to CGRP and not to placebo; adrenomedullin rate is based on [99]; nitroglycerin rate is based on [190]; cilostazol (PDE3 inhibitor) rate is based on [116]; sildenafil (PDE5 inhibitor) rate is based on [129], and levcromakalim rate is based on [137, 191]. Please note that [191] only included migraine with aura patients

Human provocation studies have contributed to an enhanced understanding of the pathophysiology of migraine and to the identification of possible new targets of treatment for migraine. Despite our growing knowledge on these alternative targets – all leading to vasodilation of intracranial arteries (Fig. 1) – no successful therapies to block CGRP-independent mechanisms have been developed yet. Indeed, an antibody to block PACAP, which is a member of the VIP, secretin, and glucagon superfamily of peptides, has been the first developed alternative therapy, although it was not effective in trials. Further, data on blockade of NO-induced responses have been ambiguous, and treatment with glibenclamide did not mitigate PACAP38-induced and levcromakalim-induced headaches. Yet, TRPV1 agonist capsaicin and civamide have shown some efficacy by their ability to desensitize nerve endings that express these channels. Further research is needed to develop alternative targeted therapies to prevent migraine (i.e. targeting VIP, amylin, adrenomedullin, PDE3, PDE5, calcium channels, and ASICs). Theoretically, blockade of the most downstream targets (e.g. K_ATP_ channels) – being the “end-chain” in the cascade – might lead to more efficacious outcomes, but also to possible severe and highly undesired side effects [189]. Whether this explains the highest induction rate of levcromakalim (Fig. 2) remains to be demonstrated.

However, to study such substances as potential candidates for migraine therapy, the presence of selective agonists or stimulating agents are indispensable. While the majority of non-CGRP targets were able to induce migraine-like attacks with relatively high induction rates (Fig. 2), this was not the case for (i) calcium channels, as it is not yet possible to directly target channels to elucidate their precise involvement in migraine; (ii) TRP channels, activation of which can induce non-migraine headache; and (iii) ASICs, as their the potential in inducing migraine attacks has not been investigated thus far. While we emphasize that the current overview is far from complete, we have aimed to provide a comprehensive overview of the (clinically) most relevant future targets for migraine. Yet, further studies on the receptor pharmacology and dual (and overlapping) agonism of several other members of the CGRP/calcitonin peptide family and calcitonin mimetics are warranted, as well as knowledge on clinical consequences of receptor internalization and recycling. As an example, salmon calcitonin was already approved as a treatment of metabolic bone diseases [192] and has been shown to have antinociceptive properties, posing it as a potential treatment migraine option as well [93, 193]. Rat studies have shown that it might ameliorate migraine-like pain through modulation of CGRP release and mast cell degranulation in the dura mater [194]. Salmon calcitonin shows potent AMY_3_ agonism, but also binds with approximately a similar affinity as human calcitonin to CTR, while amylin/pramlintide can also activate these latter receptors [93, 195].

Further, human provocation studies, in which these (non-CGRP) targets are studied through their ability to induce migraine attacks, are necessary. A limitation of these human provocation studies is that they cannot reveal the initial cause of spontaneous migraine attacks [105], nor are they able to provoke migraine auras consistently [126, 196], except for levcromakalim [137]. In addition, they do not induce attacks in all patients or account for differences in trigger sensitivity between migraine patients and the interplay of different compounds [144]. Indeed, theoretically, it should be considered that blockade of one system might lead to (over)activation of compensatory mechanisms. Therefore, the development of blockers (antagonists) of one (isolated) target or system might not guarantee efficacious headache relief in all migraine patients. Also, in different migraine patients, different pathways might be primarily activated – as demonstrated by the clinical study of Ghanizada et al. who showed that a subgroup of migraine patients only respond to CGRP and a few only to pramlintide [88]. It remains to be demonstrated whether this subgroup of patients are also non-responders to CGRP(-receptor) targeted therapies.

Future translational studies are warranted to further optimize human provocation models that allow the study of novel drug targets in migraine. In addition, basic and clinical studies are needed to improve our understanding on (i) the role of these non-CGRP targets in uncontrolled settings, given the controlled environment of the human provocation studies; (ii) the eventual role of additional targets that might play a role in the pathophysiology of auras; (iii) potential (long-term) side effects, especially considering the physiological role of e.g. adrenomedullin in the cardiovascular system [197]; (iv) the relationship between the relative contribution of different targets (e.g. amylin, adrenomedullin, PACAP) in the ictal and interictal phase in both EM and CM patients; and (v) the role of sex (steroids) on the activity of these different targets, and vice versa, considering the marked influence of sex steroids in migraine [198]. In the longer term, studies should be conducted to investigate the potential efficacy of these alternative therapies in non-responders of CGRP(-receptor) targeted therapies with the ultimate aim to, hopefully, pave the way towards a headache-free future for all migraine patients.

## Conclusion

In conclusion, the development of alternatives for CGRP(-receptor) blocking agents is essential, given the amount of non-responders to these drugs and the potential (long-term) side effects. While current research on these non-CGRP drug targets has not led to the development of efficacious therapies yet, studies on their provoking substances in human models have provided valuable insights into underlying mechanisms of migraine headaches and auras. Further research is warranted to understand the interplay of these different agents in the ictal and interictal phase as well as their relative contributions, especially in relation to the heterogeneous manifestations of migraine.

## Data Availability

Not applicable.

## Abbreviations

AC: Adenylate Cyclase
ADM: Adrenomedullin
AMP: Adenosine Monophosphate
AMY_1_: Amylin Receptor 1
AMY_2_: Amylin Receptor 2
AMY_3_: Amylin Receptor 3
ASIC: Acid-Sensing Ion Channel
BBB: Blood Brain Barrier
BK_Ca_: Big Conductance Calcium-Activated Potassium Channel
cAMP: Cyclic Adenosine Monophosphate
cGMP: Cyclic Guanosine Monophosphate
CGRP: Calcitonin Gene-Related Peptide
CLR: Calcitonin-Like Receptor
CSD: Cortical Spreading Depression
CTR: Calcitonin Receptor
FMH: Familial Hemiplegic Migraine
K_ATP_: Adenosine Triphosphate-Sensitive Potassium Channel
NO: Nitric Oxide
PAC_1_: Pituitary Adenylate Cyclase-Activating Polypeptide Type 1 Receptor
PACAP: Pituitary Adenylate Cyclase-Activating Polypeptide
PDE: Phosphodiesterase
PKA: Protein Kinase A
PKG: Protein Kinase G
RAMP1: Receptor Activity-Modifying Protein
SCA: Spinocerebellar Ataxia
sGC: Soluble Guanylyl Cyclase
TG: Trigeminal Ganglion
TNC: Trigeminal Nucleus Caudalis
TRP: Transient Receptor Potential
VIP: Vasoactive Intestinal Polypeptide
VPAC: Vasoactive Intestinal Polypeptide Receptor
